# New Appliances and Things Medical

**Published:** 1898-01-22

**Authors:** 


### NEW APPLIANCES AND THINGS MEDICAL.

**[We shall be glad to receive, at our Office, 28 & 29, Southampton Street, Strand, London, W.C., from the manufacturers, specimens of all new preparations and appliances which may be brought out from time to time.]**

### HUMANIZED MILK (SAP BRAND).

(THE MILK PREPARATIONS COMPANY, BRENTWOOD.)

The use of milk which has been prepared from cow's milk so as to agree in chemical composition with the natural milk of the mother is one of the great advances which have been made in the artificial feeding of children, who from various causes are debarred from being fed naturally. The difference between mother's milk and cow's milk, so far as chemical composition distinguishes them, consists in there being less coagulable albumenoids and more saccharine material in the former. The "Sap Brand" of Humanized Milk approximates to the chemical composition of human milk. The analysis gave 3·0 per cent. of fat, 5·2 per cent. sugar, 2·6 per cent. albumenoids, and 0 5 per cent. mineral constituents. It is contained in sterilised bottles, and the only precaution necessary is to well disseminate the cream, which always separates in sterilised milk, by agitating the bottle, after placing bottle and contents in scalding water, before using the milk.

### THE ALFORMANT.

(THE FORMALIN HYGIENIC COMPANY, LTD., 9 AND 10, ST. MARY-AT-HILL, E.C.)

Formaldehyde in its liquid form has been favourably received as a very useful antiseptic and disinfectant. Its polymer or para-formaldehyde is a solid substance, which is volatile at a gentle heat, evolving vapours of formaldehyde. It is this property of "dry formalin" (para-formaldehyde) that has been taken advantage of in the Alformant lamp. The Alformant consists of a strong metallic spirit lamp with chimney of stout glass, on the top of which is a metallic cup to contain the dry formalin tablets, ten of which suffice for every 1,000 cubic feet of room capacity. The vapours evoked from dry formalin are not unpleasant, if somewhat irritating, and are said to be quite harmless to human respiratory organs, and to have no injurious action on articles of furniture. The Alformant should be very useful as a safe and efficient means of aërial disinfection.

### FRESH FISH FOR INSTITUTIONS.

(MESSRS. SPROSTON, FISH MERCHANTS, GRIMSBY.)

There can be no doubt that when fish is required in large quantities, as at hospitals and kindred institutions, it is much better in every way to procure it direct from a fish merchant. The quality is better and the expense is far smaller. Prompt delivery can be secured by dealing with a business-like firm. Messrs. Sproston, of Grimsby, have command of a first-class supply of fish, and this they send well packed and in excellent condition at a most moderate cost.

